# Cellular Senescence Dysregulates Antiviral Interferon Responses in Idiopathic Pulmonary Fibrosis

**DOI:** 10.1111/acel.70763

**Published:** 2026-10-20

**Authors:** Jun‐Wei B. Hughes, Yasmina Reisser, Franziska Hornung, Tyler A. U. Hilsabeck, Fiona Senchyna, Ana L. Coelho, Tsung‐Che Ho, Kevin Schneider, David Furman, Cory M. Hogaboam, Claude Jourdan Le Saux, Pierre‐Yves Desprez, Stefanie Deinhardt‐Emmer

**Affiliations:** ^1^ Buck Institute for Research on Aging Novato California USA; ^2^ Leonard Davis School of Gerontology University of Southern California Los Angeles California USA; ^3^ Institute of Medical Microbiology Jena University Hospital Jena Germany; ^4^ Cedars‐Sinai Los Angeles California USA; ^5^ University of California, San Francisco San Francisco California USA; ^6^ California Pacific Medical Center Research Institute San Francisco California USA

**Keywords:** cellular senescence, idiopathic pulmonary fibrosis, influenza A virus, interferon signaling, IRF3/STAT1, lung fibroblasts

## Abstract

Patients with idiopathic pulmonary fibrosis (IPF) are highly vulnerable to respiratory virus infections, but the cellular mechanisms linking fibrotic remodeling to impaired local antiviral defense remain unclear. Here, we investigated how cellular senescence shapes the response of patient‐derived healthy (HLF) and IPF primary lung fibroblasts to influenza A virus (IAV) infection. Transcriptomic profiling identified infection as the driver of gene expression in both DNA damage‐induced senescent HLF and IPF fibroblasts and revealed induction of canonical antiviral pathways in both cell states. However, senescent IPF fibroblasts adopted a distinct antiviral response state characterized by a broader set of uniquely induced genes and differential coordination of antiviral transcriptional networks. Functionally, senescence increased viral titers in HLF and IPF fibroblasts, while senescent IPF fibroblasts displayed an altered inflammatory response. Network analysis linked viral response‐ and cell cycle‐associated modules specifically to the senescent HLF infected state, whereas these programs were weaker in senescent IPF fibroblasts. Transcription factor inference identified *IRF3* and *STAT1* as candidate regulators of this altered antiviral state in both senescent HLF and IPF fibroblasts. Consistent with the network and transcription factor analyses, siRNA‐mediated depletion of *IRF3* or *STAT1* significantly reduced IFN‐β secretion in senescent HLF fibroblasts, whereas IPF fibroblasts showed only milder effects, indicating a disease‐specific dependence on these pathways for antiviral control. Together, these findings show that the combination of cellular senescence and fibrotic fibroblast identity creates a dysfunctional antiviral state that may help explain the high susceptibility of IPF patients to virus‐associated acute exacerbations and disease worsening.

## Graphical abstract

Senescent idiopathic pulmonary fibroblasts (IPF) lung fibroblasts are transcriptomically uncoupled from a functional response to virus relative to healthy (HLF) lung fibroblasts. HLF lung fibroblasts require viral effectors STAT1 or IRF3 to enact a viral response while IPF lung fibroblasts do not rely on these axes in response to virus.

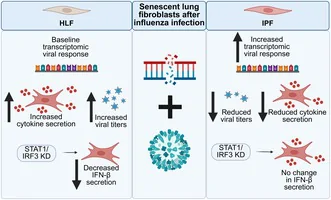

## 1 Introduction

Idiopathic pulmonary fibrosis (IPF) is a progressive, irreversible interstitial lung disease increasingly recognized as an age‐associated disorder of the lung (King et al. 2011; Martinez et al. 2017; Maher et al. 2021). In current models of IPF pathogenesis, aging is no longer regarded merely as a demographic backdrop, but as a mechanistic driver of disease. Among the hallmarks of aging, cellular senescence has emerged as a key process contributing directly to aberrant tissue remodeling (Sucre and Plosa 2019; Álvarez et al. 2017; Schafer et al. 2017; Han et al. 2023). Experimental evidence further supports a causal role of senescent cells in fibrotic lung disease, as their accumulation is detectable in fibrotic tissue and their clearance can attenuate pulmonary fibrosis in vivo (Sucre and Plosa 2019; Álvarez et al. 2017; Schafer et al. 2017; Han et al. 2023).

Respiratory infections are clinically important in IPF, where they have been implicated not only as cofactors of disease progression but also as potential triggers of acute exacerbations, the most devastating complication of the disease (Molyneaux and Maher 2013; Mostafaei et al. 2021). Acute exacerbations of IPF are associated with very poor short‐term outcomes, with reported mortality often exceeding 50% despite therapeutic intervention (Ryerson et al. 2015; Chen et al. 2024). This clinical vulnerability suggests that impaired local host defense is a relevant component of fibrotic lung disease.

Among respiratory pathogens, influenza A virus (IAV) represents a major cause of viral lower respiratory tract infection worldwide and thus provides a clinically and biologically relevant model with which to interrogate antiviral defense in the fibrotic lung. Respiratory viral infections, including influenza, have been implicated as potential triggers of acute exacerbations in IPF, further supporting the use of IAV as a model of infection‐associated disease worsening (Sese et al. 2021; Kolb and Richeldi 2011; Huang and Tang 2021).

Although epithelial injury remains central to prevailing concepts of fibrosis, fibroblasts are increasingly appreciated as immunologically competent structural cells that can sense danger signals, respond to inflammatory cues, and actively shape local innate defense programs (Cavagnero and Gallo 2022; Ghonim et al. 2023). Among the stromal compartments implicated in IPF, fibroblasts occupy a central position because they are both major effector cells of extracellular matrix deposition and active regulators of tissue remodeling (Álvarez et al. 2017; Schafer et al. 2017; Han et al. 2023). Various subtypes of fibroblasts isolated from IPF lungs display senescence‐associated phenotypes characterized by altered survival programs, resistance to apoptosis, mitochondrial dysfunction, telomere abnormalities, and acquisition of a senescence‐associated secretory phenotype (SASP), all of which are thought to reinforce profibrotic signaling within the diseased lung (Han et al. 2023; Schafer et al. 2017; Álvarez et al. 2017). However, despite substantial progress in defining fibroblast senescence as a driver of matrix remodeling, far less is known about how senescence shapes the capacity of lung fibroblasts to mount intrinsic responses to viral infection.

This question is particularly relevant in the context of IAV infection. Cell‐intrinsic defense against IAV depends on rapid sensing of viral RNA and activation of interferon‐centered transcriptional programs (Iwasaki and Pillai 2014; Opitz et al. 2007; Tolomeo et al. 2022; Talon et al. 2000). In lung cells, RNA Sensor RIG‐I (*RIG‐I*)‐dependent signaling and Interferon Regulatory Factor 3 (*IRF3*) activation are critical for induction of type I interferons, whereas downstream transcription factors such as Signal Transducer and Activator of Transcription 1 (*STAT1*) orchestrate the antiviral interferon‐stimulated gene (ISG) response (Iwasaki and Pillai 2014; Opitz et al. 2007; Tolomeo et al. 2022; Talon et al. 2000). Aging, however, has been linked to impaired or dysregulated antiviral interferon responses, including defects in *RIG‐I*‐associated signaling, raising the possibility that age‐related cellular states may uncouple viral sensing from effective effector function (Tolomeo et al. 2022; Molony et al. 2017). In the setting of influenza, fibroblasts are not passive bystanders but can adopt antiviral and pro‐inflammatory states that influence tissue pathology and repair (Ghonim et al. 2023). Whether such rewiring also occurs in senescent IPF fibroblasts remains unresolved.

We hypothesize that after infection, senescent IPF lung fibroblasts adopt an amplified, pro‐inflammatory state that associates with worsening tissue pathology and repair in IPF. Addressing this question may provide mechanistic insight into why the aging fibrotic lung is particularly susceptible to infection‐associated deterioration and acute worsening. We therefore investigated whether senescence in fibrotic fibroblasts amplifies antiviral signaling or instead drives a distinct host‐response state in which viral sensing, interferon output, and functional control of infection become uncoupled.

## 2 Results

### 2.1 Senescent Healthy and IPF Lung Fibroblasts Exposed to Infection Activate Canonical Viral Response Pathways With Heterogeneity at the Individual Gene Level

Prior to investigating differences in response to infection between senescent healthy (HLF) and IPF lung fibroblasts, we wanted to determine whether there were baseline differences in viral‐associated pathways in quiescent HLF and IPF lung fibroblasts prior to senescence or infection. We used bulk RNA sequencing (RNA‐seq) data from the quiescent HLF and IPF lung fibroblasts with mock infection from this study as well as a previously deposited but unanalyzed dataset with quiescent HLF and IPF lung fibroblasts without mock infection. In both datasets with multiple differential expression analysis methods, we observed repression of viral‐associated pathways in IPF lung fibroblasts relative to HLF, which included gene pathways associated with interferon response signaling, negative regulation of viral processes, and viral genome replication (Figure S1A). Additionally, to identify upstream regulators of the distinct antiviral states, we performed transcription factor (TF) enrichment analysis on the RNA‐seq data (Müller‐Dott et al. 2023). This approach uses perturbation‐derived datasets and has been validated in human systems (Müller‐Dott et al. 2023). In quiescent IPF lung fibroblasts, we show predicted repression of viral‐associated TFs such as IRF1, STAT1, and STAT3 relative to HLF lung fibroblasts (Figure S1B). Since senescence is already known to amplify viral response, these baseline analyses gave us confidence in our hypothesis that IPF lung fibroblasts would show increased pro‐inflammatory response to virus potentially due to an initial weakened viral response state.

To understand functional differences between senescent HLF and IPF lung fibroblasts, we exposed HLF (3 donors) and IPF (4 donors) lung fibroblasts to 15 Grays γ‐irradiation (IR), a well‐established method that triggers senescence primarily through DNA double‐strand breaks (DSBs) (Figure 1A) (Coppé et al. 2008; Schafer et al. 2017; Miller et al. 2025; Maus et al. 2023; Hernandez‐Gonzalez et al. 2023). 10 days after IR, we exposed senescent HLF and IPF lung fibroblasts to influenza A virus (IAV) infection for 24 h (h), a well‐established model for the increased prevalence of viral infections in patients with IPF (Figure 1A). A 24 h infection time point was selected based on previous results from infecting human lung fibroblasts (IMR‐90 cells) with IAV for 8 and 24 h. At 24 h, viral load was significantly increased, along with increased and broader expression of interferon‐stimulated genes (ISGs), which are central to the cellular antiviral defense, compared to 8 h (Figure S2A,B). After 24 h of infection, we collected RNA from the lung fibroblasts for RNA‐seq. Principal component analysis revealed infection largely driving gene expression changes (Figure 1B). Differential expression analysis uncovered a larger proportion of upregulated than downregulated genes in both senescent HLF and IPF lung fibroblasts after infection (Figure 1C). Senescent IPF lung fibroblasts showed more upregulated genes than senescent HLF lung fibroblasts with 176 unique, significantly upregulated genes in response to infection (Figure 1D). Shared, significant upregulated DEGs included viral response in both senescent HLF and IPF infected conditions, such as *IFNβ1* upregulation in both conditions (Figure 1E). Many upregulated genes in both senescent HLF and IPF lung fibroblasts after infection were related to viral response, as shown by Gene Ontology (GO) enrichment and gene set enrichment analysis (GSEA) (Figure 1G and Figure S3A,B). Senescence‐associated pathways also showed high levels of similarity by GSEA and notable downregulation of SASP‐associated pathways (Figure S4A,B). Interestingly, using GSEA of metabolism‐associated pathways, we show unique changes between HLF and IPF lung fibroblasts after senescence and infection (Figure S4C). Prior to senescence induction and infection, IPF lung fibroblasts showed repression of pathways associated with mitochondrial translation, respiratory electron transport, and oxidative phosphorylation (Figure S4C). After senescence induction and infection, this decreased enrichment remained the same in IPF lung fibroblasts while HLF lung fibroblasts showed enrichment for these pathways (Figure S4C). In sum, while virus‐associated and senescence‐associated pathways showed largely similar enrichment between senescent HLF and IPF lung fibroblasts after infection, metabolism‐related pathways were unique between HLF and IPF lung fibroblasts.

**FIGURE 1:**
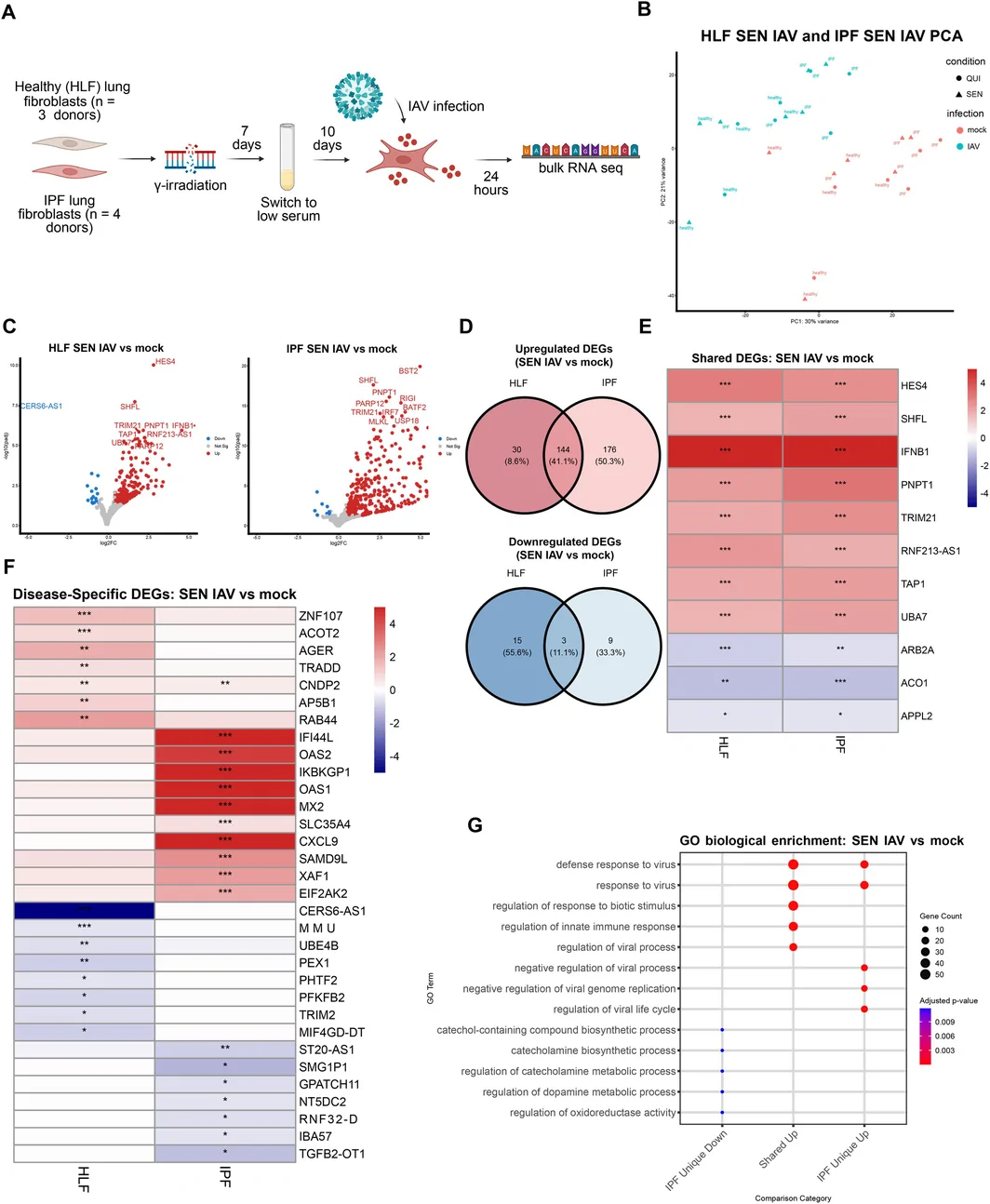
Senescent healthy and IPF lung fibroblasts exposed to infection activate canonical viral response pathways with heterogeneity at the individual gene level. (A) Schematic of 3 healthy (HLF) and 4 idiopathic pulmonary fibrosis (IPF) primary, human, lung fibroblasts, between ages 37–61, induced to senesce by γ‐irradiation (IR; SEN). Quiescent (QUI) lung fibroblasts were changed to low serum (LS) 3 days prior to infection. Influenza A virus (IAV) infection was performed at 10 days after IR for 24 h and RNA was collected for bulk RNA sequencing (RNA‐seq) after the 24 h infection period. (B) Principal component analysis (PCA) of QUI and SEN HLF and IPF lung fibroblasts with mock or IAV treatment. (C) Volcano plot of differentially expressed genes (DEGs) in HLF SEN IAV (left) vs. IPF SEN IAV (right) lung fibroblasts. DEG cutoffs were defined by log_2_FC < −0.5 or > 0.5 and *p*‐adjusted value < 0.05. Top 20 DEGs labeled and the volcano plot was capped at −5 to 5 log2FC. (D) Venn diagram of shared and unique upregulated (top) and downregulated (bottom) DEGs between HLF SEN IAV vs. IPF SEN IAV lung fibroblasts. (E) Heatmap of shared top 10 upregulated and downregulated DEGs between HLF SEN IAV vs. IPF SEN IAV lung fibroblasts. (F) Heatmap of unique top 10 upregulated and downregulated DEGs between HLF SEN IAV vs. IPF SEN IAV lung fibroblasts. (G) Top 5 GO enrichment terms from HLF and IPF SEN IAV uniquely upregulated and downregulated genes and HLF and IPF SEN IAV shared upregulated and downregulated genes. Only 4 categories of GO enrichment shown as HLF and IPF SEN IAV shared downregulated genes and HLF SEN IAV uniquely upregulated genes did not enrich for GO terms.

Further differences between HLF and IPF lung fibroblasts were uncovered when analyzing important viral‐associated pathways at the individual gene level. Antiviral effector genes, such as 2′‐5′‐oligoadenylate synthetase 1 and 2 (*OAS1* and *OAS2*), myxovirus resistance‐1 (*MX1*), radical S‐Adenosyl Methionine Domain Containing 2 (*RSAD2*), and chemokine ligand 10 (*CXCL10*), were all significantly upregulated only in the senescent IPF infected condition (Figures 1F and S3B). Interestingly, *OAS1* was significantly downregulated in quiescent IPF lung fibroblasts prior to senescence induction and infection, potentially indicating a compensatory antiviral response (Figure S3B). Overall, senescent HLF and IPF lung fibroblasts exhibit similar responses to influenza infection at the global level with a seemingly amplified response in IPF lung fibroblasts at the level of individual genes.

### 2.2 Senescence in IPF Fibroblasts Results in Decreased Viral Titers and Altered Cytokine Production

To further investigate the impact of senescence on IPF fibroblasts in the context of viral infections, quiescent and senescent HLF and IPF fibroblasts were infected with IAV at a multiplicity of infection (MOI) of 1 for 24 h (Figure 2A). Specifically, changes in viral titers and cytokine release were analyzed. In both HLF and IPF conditions, senescent fibroblasts displayed an increased virus titer compared to the respective quiescent control fibroblasts (Figure 2B). Unexpectedly, the virus titer in senescent IPF fibroblasts was significantly lower than the virus titer in senescent HLF fibroblasts 24 h post infection (hpi). Interestingly, quiescent IPF fibroblasts showed a modest, nonsignificant decrease in viral titer relative to quiescent HLF fibroblasts. Quantification of intracellular viral RNA at 24 hpi showed a similar pattern to that observed for viral titers in the supernatant (Figure S5). These findings imply a potential association between diminished viral replication and senescence in the IPF fibroblasts. A multiplex cytokine assay of the supernatant of mock and infected fibroblasts revealed a general trend to reduced pro‐inflammatory cytokine production in infected quiescent and senescent IPF fibroblasts (Figure 2C). Specifically, IP‐10 (*CXCL10*), critical in the inflammatory response to IAV, was significantly lower in both quiescent and senescent IPF fibroblasts, indicating a modified response of these cells to IAV infection. Overall, our findings suggest that senescence induction in IPF lung fibroblasts results in a transformed reaction to viral infections, characterized by both diminished viral replication and reduced cytokine secretion.

**FIGURE 2:**
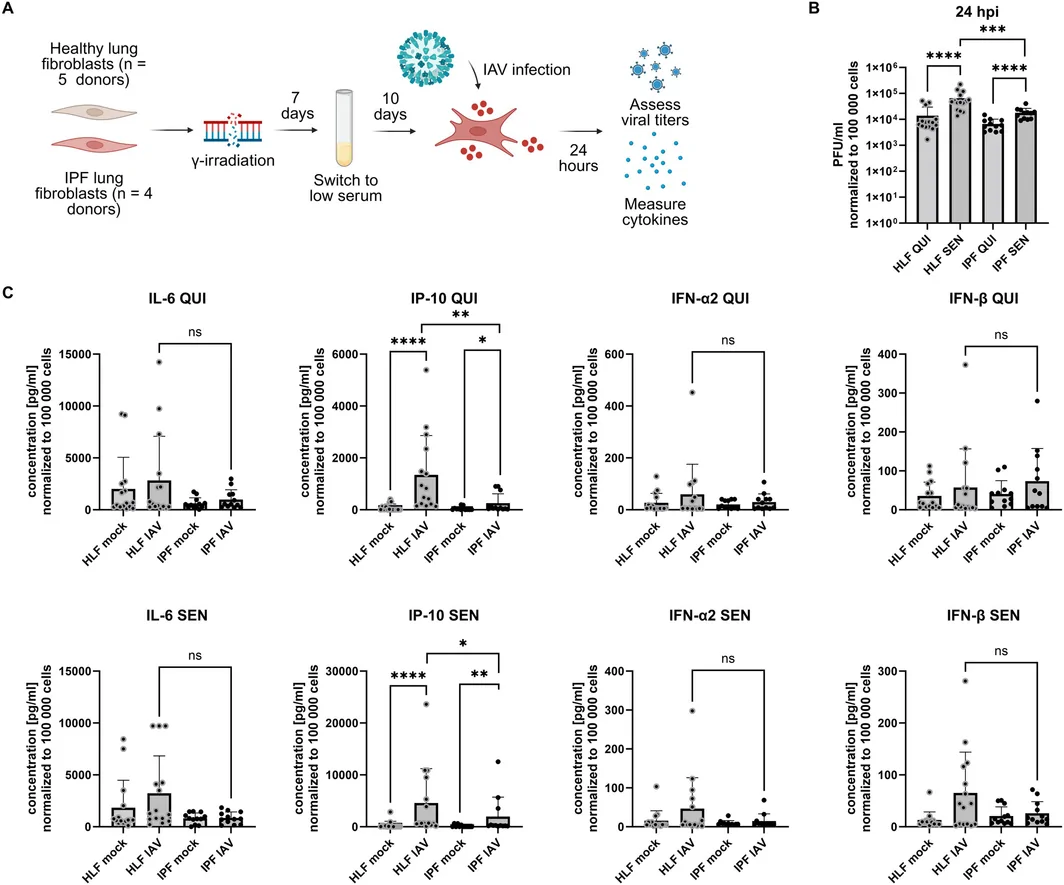
Senescence in IPF fibroblasts results in decreased viral titers and altered cytokine production. (A) Schematic overview of quiescence and senescence induction and subsequent viral infection. (B) Viral titers determined by plaque assay 24 h post infection (hpi) (healthy lung fibroblasts (HLF): 5 donors with each 3 replicates, idiopathic pulmonary fibrosis (IPF) fibroblasts: 4 donors with each 3 replicates). Plaque‐forming units (PFU) have been normalized to cell count and are displayed as PFU/ml adjusted to 100,000 cells. Data is displayed on a logarithmic scale. (C) Quantification of cytokines (IL‐6, IP‐10, IFN‐ɑ2, and IFN‐β) 24 hpi in the supernatant of mock and infected quiescent (QUI) and senescent (SEN) HLF as well as IPF cells (HLF: 5 donors with each 3 replicates, IPF: 4 donors with each 3 replicates). Cytokine concentration has been normalized to the cell count and is shown as concentration in pg/ml adjusted to 100,000 cells. Statistical significance was calculated using the Mann–Whitney U test (B, C). *p < 0.05; **p < 0.01; ***p < 0.001; ****p < 0.0001.

### 2.3 Senescent Healthy Lung Fibroblast Gene Expression Signature Is Connected to Viral Responses and the Cell Cycle

We next wanted to uncover targets driving the lack of functional response to infection in senescent IPF lung fibroblasts. To do this, we used weighted gene correlation network analysis (WGCNA), which generates correlation networks separate from differential expression analysis, to uncover gene and gene networks driving the senescent IPF infected cell state (Figure 3A) (Langfelder and Horvath 2008). The WGCNA revealed two significantly correlated modules, Black and Brown, to the senescent HLF infected condition that were not significantly correlated to the senescent IPF infected condition (Figure 3B). The Black module genes were enriched for genes associated with viral response, while the Brown module enriched for genes associated with the cell cycle (Figure 3C). This seems consistent with our differential expression and functional analyses, as the senescent IPF lung fibroblasts upregulate more genes related to viral response but fail to functionally respond to a viral infection, while senescent HLF lung fibroblasts have a stronger connected response to viral infection, as shown functionally and by the WGCNA. Individual genes in each module, such as retinoic acid‐inducible gene I (*RIGI*) in the Black module, which detects viral RNA inside infected cells and is known to be impaired in various aging contexts, show potential targets driving the senescent HLF infected cell state and reinforce our differential expression and functional analyses (Figure 3D) (Molony et al. 2017).

**FIGURE 3:**
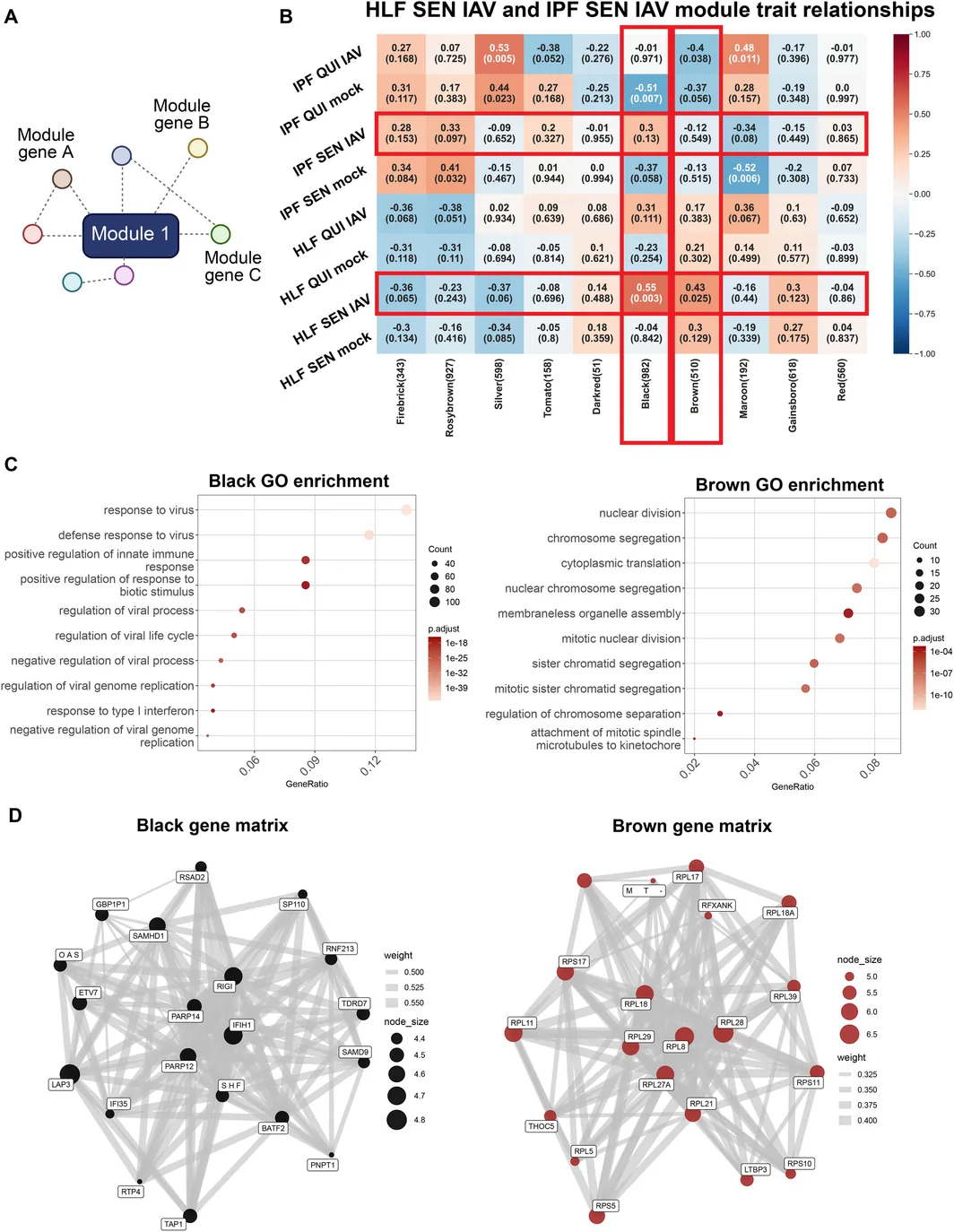
Senescent healthy lung fibroblast gene expression signature is strongly connected to viral responses and the cell cycle. (A) Schematic of WGCNA module connections between genes within a module. (B) WGCNA module‐trait heatmap relationships. The rows are the conditions (disease, condition, infection) and each column is a module eigengene. Each cell includes corresponding correlation to the specific module and correlation is indicated by the value in the box. Correlation ranges from −1 to 1, and blue indicates a negative correlation while red indicates a positive correlation. Correlation values are above the number in parenthesis which indicates the *p* value of each module's connectivity. Rectangular red boxes are drawn around intersecting rows and columns of interest. HLF = healthy lung fibroblasts. (C) Top 10 GO enrichment terms of genes from the Black (left) and Brown (right) modules. (D) Module gene network visualization for the Black (left) and Brown (right) modules. Nodes represent the top module genes ranked by connectivity z‐score, node size scales with intramodular connectivity, and edges represent weighted topological overlap matrix (TOM) connections (TOM ≥ 0.001; top 8 connections retained per gene).

### 2.4 Transcription Factor Inference Identifies Differential IRF3/STAT1‐Associated Antiviral Programs in Senescent Healthy and IPF Lung Fibroblasts

To identify upstream regulators of the distinct antiviral states after senescence induction in HLF and IPF lung fibroblasts, we again performed transcription factor (TF) enrichment analysis on the RNA‐seq data (Müller‐Dott et al. 2023). The TF prediction analysis showed a broadly similar set of virus‐associated TFs in senescent HLF and IPF fibroblasts after infection (Figure 4A). However, the inferred level of activation in senescent IPF lung fibroblasts after infection was higher for virus‐associated TFs, such as *STAT1* and *IRF3*, than in HLF lung fibroblasts (Figure 4A). *IRF3* became of particular interest because this TF has been less well studied in the context of senescence, fibrosis, and viral infection. *STAT1* was also highlighted because it is a well‐established regulator of antiviral interferon responses (Figure 4A). Both *STAT1* and *IRF3* showed an increase of about 5 in activity score in senescent IPF lung fibroblasts relative to HLF (Figure 4A). To support these predictions, we mapped the individual genes contributing to each TF‐associated signature. This revealed strong concordance between inferred TF activity and the underlying gene expression patterns in both conditions (Figure 4B). Overall, this data suggests that senescent HLF and IPF fibroblasts engage a similar antiviral TF repertoire after infection but differ in the relative activation of key interferon‐associated regulators, particularly *IRF3* and *STAT1*.

**FIGURE 4:**
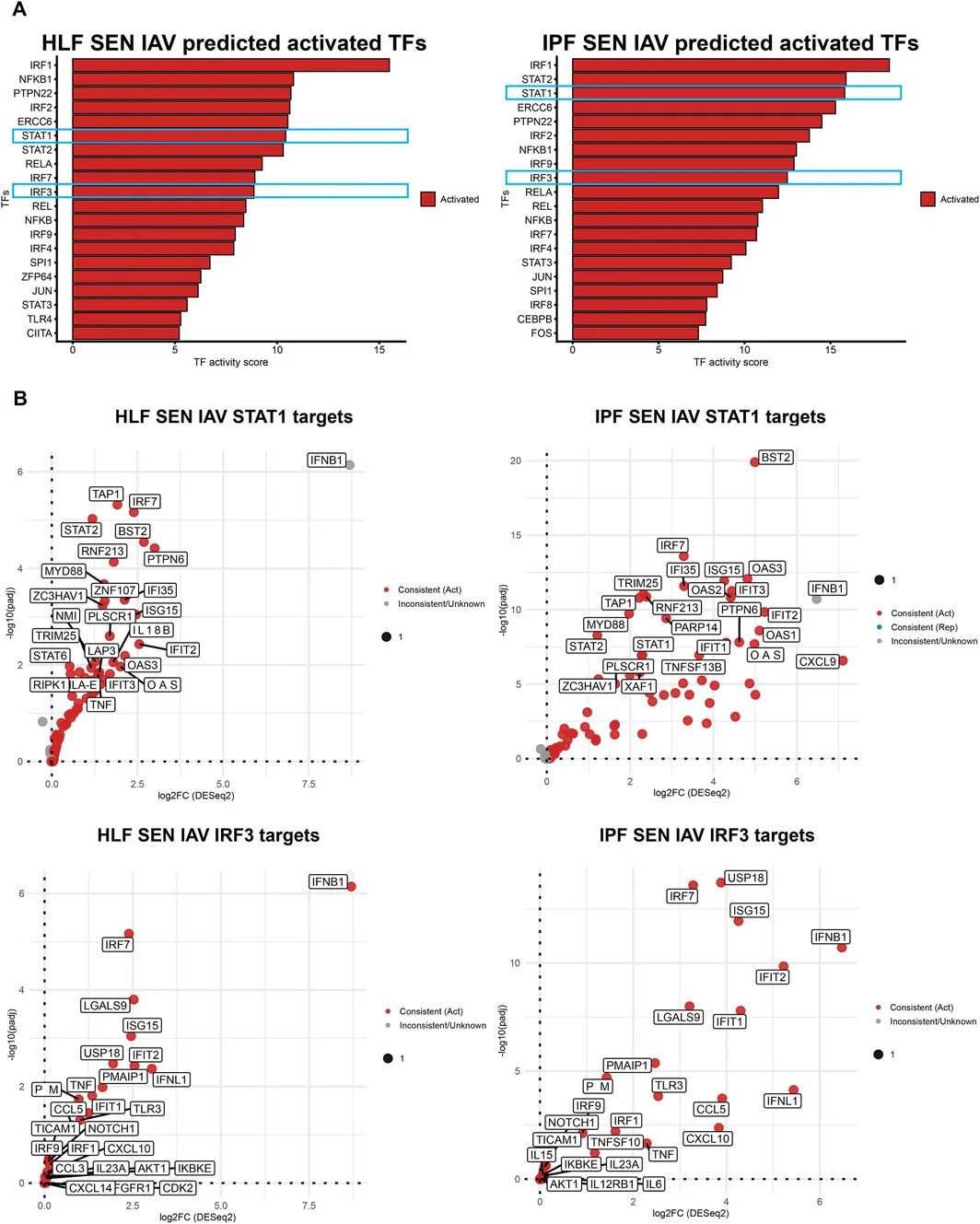
Transcription factor inference identifies differential IRF3/STAT1‐associated antiviral programs in senescent healthy and IPF lung fibroblasts. (A) Top activated transcription factors (TF) inferred by decoupleR (ULM) using CollecTRI and DESeq2 Wald statistics for healthy (HLF) SEN IAV (left) or IPF SEN IAV lung fibroblasts (right). Bars indicate TF activity scores (red, activated; blue, repressed). No repressed TFs were predicted for either comparison. TFs of interest highlighted in rectangular blue boxes. (B) HLF SEN IAV (left panels) and IPF SEN IAV (right panels) CollecTRI target genes for *STAT1* and *IRF3*, with points colored by concordance (red: Consistent with predicted activation, blue: Consistent with predicted repression, gray: Inconsistent with prediction) between predicted TF regulation and observed RNA‐seq differential expression.

### 2.5 IRF3 And STAT1 Are Required for Antiviral Control in Senescent Healthy Fibroblasts

TF analysis identified *IRF3* and *STAT1* as candidate regulators of the altered antiviral state in infected senescent lung fibroblasts. To test their functional contribution, quiescent and senescent HLF and IPF fibroblasts were transfected with non‐targeting (NT), *IRF3*, or *STAT1* siRNA 24 h prior to IAV infection (Figure 5A). Downregulation of mRNA expression level confirmed efficient knockdown of both *IRF3* and *STAT1* across all conditions (Figure 5B). For IRF3 and STAT1, a modest reduction at the protein level was additionally verified in senescent HLF fibroblasts (Figure S6A,B). While mock cells showed only very low levels of p‐STAT1, with a strong increase in IAV infected samples, mock cells transfected with *STAT1* siRNA also showed a slight reduction in p‐STAT1 levels. In cells transfected with *IRF3* siRNA, however, no clear reduction in p‐IRF3 was detected in the evaluated samples. However, phosphorylation levels of IRF3 were generally low across all conditions, suggesting that the modest reduction in unphosphorylated IRF3 remains functionally relevant in this model.

**FIGURE 5:**
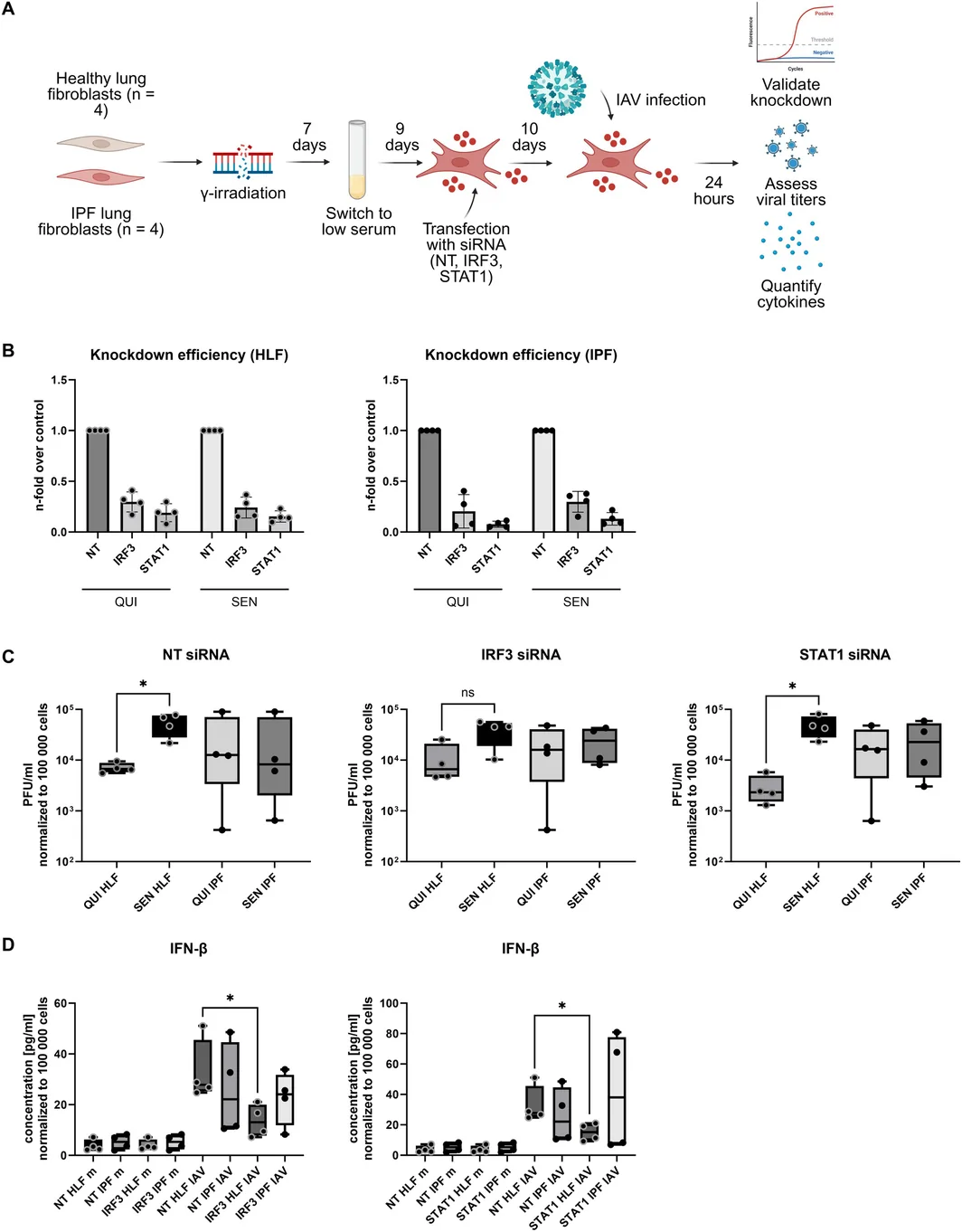
IRF3 and STAT1 are required for antiviral control in senescent healthy fibroblasts. (A) Schematic overview of transfection workflow of quiescent (QUI) and senescent (SEN) healthy lung fibroblasts (HLF) and idiopathic pulmonary fibrosis (IPF) fibroblasts with Accell siRNA (NT, *IRF3* and *STAT1*) 24 h prior to infection. (B) Assessment of knockdown efficiency for *IRF3* and *STAT1* knockdown on expression level via qRT‐PCR (*n* = 4). Expression is shown as fold change relative to the control NT siRNA condition. (C) Viral titers 24 h post infection (hpi) in all transfected conditions determined via plaque assay (*n* = 4). PFU has been normalized to cell count and is displayed as PFU/ml adjusted to 100,000 cells. Data is displayed on a logarithmic scale. (D) Cytokine quantification of IFN‐β in supernatants from SEN HLF and IPF fibroblasts transfected with NT, *IRF3* (left), or *STAT1* (right) siRNA under mock or infection conditions at 24 hpi. Cytokine concentration has been normalized to cell count and is displayed as concentration in pg/ml adjusted to 100,000 cells. Statistical significance was calculated using Mann–Whitney U test (C, D). *p < 0.05.

Functional perturbation revealed that senescent HLF fibroblasts were partially dependent on *IRF3*/*STAT1* signaling for antiviral control. In these fibroblasts, *IRF3* knockdown was accompanied by a lack of increase in viral plaque formation at 24 hpi (Figure 5C). Additionally, depletion of either *IRF3* or *STAT1* significantly reduced IFN‐β secretion after infection in senescent HLF fibroblasts (Figure 5D). In contrast, senescent IPF fibroblasts showed only milder changes following knockdown of *IRF3* or *STAT1*, indicating that the antiviral response of senescent HLF fibroblasts relies more strongly on these signaling nodes than the corresponding response in senescent IPF fibroblasts. Thus, despite retaining transcriptional evidence of antiviral pathway activation, senescent IPF fibroblasts appear more independent of *IRF3*/*STAT1* interferon signaling after infection relative to senescent HLF fibroblasts.

To determine whether siRNA transfection influenced senescence‐associated cell state stability, expression of *CDKN1A*, *CDKN2A*, and *LMNB1* was assessed in transfected fibroblasts (Figure S6C). Although variability in senescence marker expression was observed after transfection, *CDKN1A* increased and *LMNB1* decreased in senescent HLF fibroblasts with *IRF3* knockdown. In contrast, in senescent IPF fibroblasts, only a significant downregulation of *LMNB1* was identified, which was exclusively observed under the non‐targeting (NT) condition, indicating alterations of the senescence phenotype in *IRF3*‐transfected IPF cells. This data supports the overall functional model in which residual antiviral control in senescent IPF fibroblasts depends less on *IRF3*/*STAT1* signaling after infection relative to senescent HLF fibroblasts.

To elucidate further differences in the antiviral response, expression of ISGs was assessed via qRT‐PCR (Figure S7A–D). *MX1* and *OAS1* were upregulated upon infection in senescent IPF fibroblasts compared to senescent HLF controls, while *IFIT1* was increased in NT transfected senescent samples and in quiescent samples with *IRF3* knockdown. No significant differences were observed for *ISG15*. Overall, ISG upregulation appeared largely independent of *IRF3* and *STAT1* knockdown. This distinct antiviral response at the individual gene level between senescent IPF and HLF samples was also reflected in the overall expression pattern observed in the RNA sequencing analysis of untransfected samples, potentially attributable to differences in baseline antiviral gene expression (Figure S3B).

Together, these findings provide functional validation of the transcription factor inference analysis and identify *IRF3*/*STAT1* as key effectors of antiviral defense in senescent HLF lung fibroblasts that are not functionally activated in IPF lung fibroblasts.

## 3 Discussion

Cellular senescence is increasingly recognized as a central feature of the aging and fibrotic lung, yet its consequences for intrinsic antiviral defense in stromal cells have remained poorly defined. In our study on senescent IPF lung fibroblasts, contrary to our hypothesis that senescent IPF lung fibroblasts would exhibit a heightened inflammatory activation, senescent IPF fibroblasts adopt an antiviral response state that is transcriptionally but not functionally engaged. Prior to infection and senescence induction, viral programs are reduced in IPF lung fibroblasts, raising the hypothesis that an initially dampened antiviral cell state would lead to a compensatory, strong induction of viral programs due to increased viral load and invasion. Instead, we observed that canonical virus‐response pathways are still induced after IAV infection but their downstream translation into coordinated antiviral control and inflammatory output is diminished and likely altered by the combination of senescence and fibrotic cell identity. This distinction is important because it suggests that fibrotic fibroblasts are not immunologically silent, but instead trapped in a maladaptive response state that is transcriptionally but not functionally active.

One of the central observations is that activation of antiviral transcriptional programs does not necessarily equate to effective antiviral defense. Both senescent HLF and senescent IPF fibroblasts upregulated virus‐associated pathways after infection, indicating that elements of pathogen sensing remain intact in both contexts. At the same time, senescent IPF fibroblasts showed a broader set of uniquely induced genes, including *OAS* family members, together with a predicted, stronger coordination of antiviral transcriptional networks. These data support a scenario in which viral sensing is initiated, but the response is not integrated into a coherent protective program, which is additionally supported by the lack of network connections associated with virus response from the WGCNA. Clinically, this could coincide with acute exacerbations leading to a detrimental, positive feedback loop and ultimately, functional lung decline. Such a state is consistent with broader aging biology, in which cellular stress responses are present but become uncoupled from initiating the appropriate effector function (Haigis and Yankner 2010; Lavretsky and Newhouse 2012; Vitlic et al. 2014; Hughes et al. 2026).

Our study shows that senescence increases the viral titers relative to quiescent controls in both HLF and IPF conditions, supporting the idea that senescent cell states can promote viral permissiveness (Schulz et al. 2023). However, senescent IPF fibroblasts displayed an altered inflammatory profile, including reduced *CXCL10*/IP‐10 secretion, despite retaining transcriptional evidence of antiviral pathway activation and specifically *CXCL10* gene upregulation. This dissociation between transcriptional induction and inflammatory output suggests that fibrotic fibroblasts fail to properly convert viral recognition into an appropriately coordinated response. In the context of the lung, fibroblasts are now more commonly known to be immunologically competent stromal cells capable of sensing danger and influencing epithelial and immune‐cell behavior (Cavagnero and Gallo 2022; Ghonim et al. 2023). Furthermore, lung fibroblasts play an important role in influenza‐driven fibrotic remodeling of the lung, placing them at the intersection of antiviral response, senescence, and fibrosis, and making them a relevant model for investigating these interactions (Reisser et al. 2026). A dysfunctional antiviral fibroblast state could then contribute to altered host defense and heightened susceptibility to infection‐associated deterioration, for instance through delayed recruitment of immune cells and subsequent prolonged persistence of IAV. As influenza infection has been shown to trigger acute exacerbations in IPF, this impaired viral clearance could further worsen disease outcome through prolonged injury of pulmonary cells or increased secretion of pro‐fibrotic factors such as TGF‐β (Huang and Tang 2021).

The network‐level analyses build on this model further. Viral‐response and cell‐cycle modules were specifically linked to the senescent HLF infected state, whereas comparable coordinated programs were weak or absent in senescent IPF fibroblasts. This finding suggests that fibrotic identity destabilizes the response to viral challenge under senescent conditions. Efficient antiviral defense requires synchronized coupling of sensing, interferon induction, and downstream effector programs. Once compromised, the resulting state may be observed as “antiviral” at the transcript level but fail at the functional level, which requires systems level coordination to enact a functional response. The WGCNA results provide an important systems level view between the transcriptomic data and functional phenotype. Although virus‐associated genes are activated in senescent IPF lung fibroblasts after infection, the response is uncoordinated within gene networks based on the WGCNA results. This is then associated with a lack of a functional response to viral infection in senescent IPF lung fibroblasts and ultimately supports the view that senescent IPF fibroblasts occupy a partially activated yet biologically incompetent antiviral state.

In the context of senescence, the SASP can influence a variety of antiviral responses. In aged mice, senescent cells secrete SASP mediators including IL‐6 and IL‐1α that collectively dampen type I interferon and ISG induction by impairing phosphorylation and activation of IRF3 and STAT1 (Zhang et al. 2026). The SASP also contributes to antiviral defense by senescent cells in vitro, with factors such as IL‐6, IL‐8, and MCP‐1 enhancing recruitment of immune cells to clear infected cells (Kelley et al. 2020; Cao et al. 2025). From our results, it seems that the SASP is being dampened to increase antiviral responses in response to infection in both HLF and IPF conditions. However, this is only inferred from gene expression results and should be verified at the protein level by quantifying secretion of SASP‐associated proteins. Additionally, given the altered host defense of senescent IPF fibroblasts and that SASP composition can be highly heterogeneous, SASP‐related gene versus protein expression could be different between HLF and IPF samples (Hernandez‐Segura et al. 2017; Basisty et al. 2020).

Senescence can also alter metabolism, including changes in mitochondrial function, glycolysis, and nutrient‐sensing pathways. Prior to and after senescence induction and infection, IPF lung fibroblasts showed depletion of oxidative phosphorylation, mitochondrial translation, and respiratory electron‐transport programs relative to HLF lung fibroblasts. This raises the possibility that senescence imposes a greater metabolic constraint on IPF fibroblasts, potentially limiting their capacity to translate proteins in response to infection despite robust antiviral transcription. Future studies should investigate and knockout specific proteins involved in these mitochondrial‐related pathways to mechanistically determine if functional antiviral activation is regulated by changes in metabolism after senescence induction.

*IRF3* and *STAT1* emerged as key regulatory nodes within this altered antiviral landscape. Although both transcription factors are central mediators of interferon signaling and were predicted to be activated in infected fibroblasts, their functional relevance differed by cellular context. In senescent IPF fibroblasts, poor coordination of the broader antiviral network may decrease reliance on core regulatory nodes, rendering antiviral control unresponsive to specific signaling axes such as through *IRF3* or *STAT1*. This continues to support the idea that although virus‐associated TFs are predicted to be activated in senescent IPF fibroblasts, the appropriate effector function is not initiated. Validating which TFs are functionally active or repressed in senescent IPF fibroblasts would help to understand at which point senescent IPF viral response capabilities could be restored.

This model offers a plausible explanation for the clinical vulnerability of patients with IPF to respiratory viral infections. Acute exacerbations are among the most devastating complications of IPF, and infections are widely considered important triggers or amplifiers of these episodes (Ryerson et al. 2015). A fibroblast compartment that retains partial viral sensing yet fails to mount a coordinated interferon‐driven response may contribute to disease worsening by blunting paracrine immune signaling and disturbing the balance between injury and repair in an already damaged lung. The use of primary human HLF and IPF fibroblasts further strengthens the clinical relevance of this concept.

One limitation of our study is the use of a single mode of senescence induction. Various senescence triggers, such as oncogene‐induced and mitochondrial dysfunction‐induced senescence, can produce distinct senescent phenotypes, and further studies are needed to evaluate how these varying induction types contribute to differences in the antiviral response (Hernandez‐Segura et al. 2017). An additional limitation is that protein level confirmation of the knockdown was performed in only two senescent HLF replicates, due to limited cell availability from other conditions. In these samples, p‐IRF3 downregulation was inconsistent, potentially reflecting SASP‐related effects on the cellular antiviral response (Zhang et al. 2026).

Overall, contrary to our hypothesis, cellular senescence does not simply intensify pre‐existing fibrotic programs. Rather, senescent IPF lung fibroblasts show a distinct form of stromal antiviral dysfunction in which canonical sensing pathways remain inducible, but their downstream execution is functionally disabled. This view places fibroblasts more centrally into the biology of infection susceptibility in the aging fibrotic lung and suggests that therapeutic strategies aimed at restoring coordinated interferon responsiveness may be more relevant than indiscriminately amplifying inflammation.

Taken together, these findings define senescent IPF fibroblasts as a dysfunctional antiviral stromal state shaped by cellular senescence and the fibrotic cell identity. Preserved elements of viral sensing coexist with impaired systems‐level coordination and altered dependency on *IRF3*/*STAT1*‐associated interferon signaling. This provides a mechanistic framework linking fibrotic remodeling to impaired local antiviral defense and may help explain why the aging fibrotic lung is particularly vulnerable to virus‐associated acute exacerbations and disease worsening.

In conclusion, our findings define senescent IPF fibroblasts as a dysfunctional antiviral fibroblast state characterized by preserved viral sensing but impaired interferon response. By identifying *IRF3*/*STAT1*‐associated signaling as a critical, missing control axis in the context of IPF lung fibroblasts, this work also highlights a potential point of therapeutic approaches in the aging fibrotic lung.

## 4 Experimental Procedures

### 4.1 Virus Strain and Propagation

Viral infections were carried out with Influenza A virus/H1N1/Puerto Rico/8/1934 (IAV), which has been propagated in Madin‐Darby canine kidney (MDCK) cells cultivated in minimum essential medium (MEM) containing 10% fetal bovine serum (FBS) and 1% Penicillin/Streptomycin (P/S) (Hornung et al. 2025). Viral titers from both propagated virus and in vitro infections were quantified as plaque‐forming units (PFU) per milliliter using a plaque assay, as previously detailed (Deinhardt‐Emmer et al. 2021). For viral titers obtained from in vitro experiments, PFU counts were normalized to cell counts and expressed as PFU per 100,000 cells.

### 4.2 Cultivation of Primary Human Lung Fibroblasts

Healthy lung fibroblasts (HLF) and lung fibroblasts from donors diagnosed with idiopathic pulmonary fibrosis (IPF) were obtained from adult donors from collaborators at the University of California, San Francisco, or Cedars‐Sinai and isolated at their originating institutions through methods previously described (Hogaboam et al. 2005). A complete list of details for each donor lung fibroblast can be found in “Table S1.” Human lung fibroblasts (IMR‐90 cells) were obtained from the Coriell Institute for Medical Research (Camden, USA). Cells were cultured in Dulbecco's Modified Eagle Medium (DMEM) supplemented with 10% FBS and 1% P/S at 37°C with 5% CO_2_.

### 4.3 Induction of Cellular Senescence

Senescence in lung fibroblasts was induced by irradiation, in which the cells were exposed to a dose of 15 Grays (Gy) while maintained in cell culture plates. Immediately after irradiation, the medium was replaced with fresh DMEM containing 10% FCS and 1% P/S. Subsequently, the medium was refreshed every 2–3 days. The induction of senescence was completed after 10 days of culture, and the senescent cells were then utilized for further experiments.

As a control, quiescent cells were generated by serum starvation as previously described (Schulz et al. 2023; Neri et al. 2021).

### 4.4 In Vitro Infections

HLF and IPF as well as IMR‐90 cells were infected with IAV at a multiplicity of infection (MOI) of 1 in Dulbecco's Phosphate Buffered Saline (DPBS) containing 0.2% bovine serum albumin (BSA; Roth, Karlsruhe, Germany), 1 mM MgCl_2_ (Sigma Aldrich), and 0.9 mM CaCl_2_ (Sigma Aldrich) for a duration of 30 min (min). Following this incubation, the cells were washed and subsequently cultured in DMEM supplemented with 0.2% BSA, 1 mM MgCl_2_, 0.9 mM CaCl_2_, and 30 ng/mL of TPCK‐treated trypsin (Thermo Fisher Scientific, Waltham, MA, USA) for 24 h (h) (Schulz et al. 2023).

### 4.5 RNA Extraction

Cells were first lysed with RLT buffer before RNA extraction was conducted using the RNeasy Mini Kit (Qiagen, Hilden, Germany) according to the manufacturer's instructions. Concentration and purity of extracted RNA were assessed with the NanoDrop spectrophotometer ND‐1000 (Thermo Fisher Scientific/PeqLab).

### 4.6 mRNA Sequencing

Library construction and total mRNA sequencing of quiescent as well as senescent mock and infected HLF (each *n* = 3) and IPF cells (each *n* = 4), as well as mock and infected IMR‐90 cells (each *n* = 3), was carried out by Novogene Co. LTD (Munich, Germany). For the IMR‐90 cell samples, Novogene additionally performed the bioinformatic analysis.

### 4.7 cDNA Synthesis and qRT‐PCR

RNA was reverse transcribed to cDNA using the High‐Capacity cDNA Kit (Thermo Fisher Scientific). qRT‐PCR was subsequently performed with the Maxima SYBR Green qPCR Master Mix (Thermo Fisher Scientific) on a Rotor Gene Q (Qiagen) as previously described (Schulz et al. 2023). Primers were purchased from metabion (Planegg, Germany) and sequences are detailed in “Table S2.” Genes of interest were normalized to β‐Actin, and the 2^−ΔΔCT^ method was employed to quantify relative gene expression.

RNA was extracted from cell samples as described above, and viral RNA was quantified by qRT‐PCR using the RIDAGENE Flu kit (R‐Biopharm AG, Darmstadt, Germany) on a Rotor‐Gene Q, according to the manufacturer's instructions.

### 4.8 Cytokine Quantification

Multiplex cytokine quantification in the supernatant of HLF and IPF fibroblasts was performed using the LEGENDplex Hu Anti‐Virus Response Panel 1 V02 (Biolegend, San Diego, CA, USA) according to the manufacturer's protocol. Measurements were conducted with the FACS Symphony A1 (BD, Franklin Lakes, NJ, USA), and data was analyzed via the Legendplex analysis software Qognit. The concentration of cytokines was normalized to the cell count and adjusted to 100,000 cells.

### 4.9 Transfection With Accell siRNA

Quiescent and senescent HLF and IPF cells were transfected 24 h prior to infection. To successfully knock down the target genes *IRF3* and *STAT1*, a method combining the transfection reagent DharmaFECT3 (Dharmacon, Lafayette, CO, USA) and Accell siRNA (Dharmacon) was adapted (Dong et al. 2020). For siRNA transfection, quiescent and senescent HLF and IPF cells were cultured in DMEM without supplements for 2 h before transfection. A siRNA pool consisting of four individual siRNAs targeting either *IRF3* or *STAT1*, along with a non‐targeting control (NT) siRNA, was utilized (*IRF3*: E‐006875‐00‐0020; *STAT1*: E‐003543‐00‐0020; NT: D‐001910‐10‐50; all Dharmacon). siRNAs were diluted in Accell delivery medium to a final concentration of 40 nM per well and were incubated at room temperature (RT) for 5 min. Simultaneously, DharmaFECT 3 transfection reagent was diluted in Accell delivery medium (0.5 μL for 24‐well plates or 1 μL for 12‐well plates) and also incubated for 5 min at RT. The diluted siRNA and DharmaFECT 3 solutions were then combined at a 1:1 ratio and further incubated at RT for an additional 20 min.

After adding 100 μL to 24‐well plates or 200 μL to 12‐well plates, the transfected cells were cultivated for 24 h at 37°C in 5% CO_2_. Transfection efficiency was assessed by qRT‐PCR to measure the reduction in target gene expression.

### 4.10 Bulk RNA‐Seq Processing

Raw reads were aligned locally using the nf‐core/rnaseq salmon pipeline in windows system linux, performing pseudo‐alignment against a standard reference transcriptome for *Homo sapiens* (GRCh38 genome) (Ewels et al. 2020; Patro et al. 2017). Transcript‐level count estimates (estimated counts and TPMs) per sample were then summarized to gene‐level count matrices using the corresponding transcript‐to‐gene annotation in R. The gene‐level estimated count matrices along with sample metadata were used for bulk RNA‐seq dataset generation and analyses. Sample metadata can be found in “Table S3.”

### 4.11 Differential Expression Analysis

DESeq2 formula used is provided here for the lung fibroblasts: design = ~batch + disease * infection * condition (Love et al. 2014). Principal component analysis (PCA) provided in Figures 1B and S8 shows the rationale for the DESeq2 formula. Infection and batch seem to be the primary drivers of variance in the PCA; however, batch corrected versus non‐batch corrected results in Figure S8 showed similar results suggesting infection was the largest driver of variance. Therefore, the non‐batched PCA was shown in Figure 1B but batched DESeq2 results were shown throughout the rest of the manuscript. GO and KEGG enrichment performed as previously described (Consortium TGO et al. 2025; Ashburner et al. 2000; Kanehisa and Goto 2000). Transcription factor (TF) enrichment was performed using the Collection of Transcription Regulation Interactions meta‐resource that was recently sign‐curated for activation or repression of each TF in the database (Müller‐Dott et al. 2023). Differentially expressed genes (DEG) lists can be found in “Data S1 and S4.”

Figure S1A used a previously deposited dataset from GEO Super Series GSE328437 RNA‐seq data GSE328392 that required a modified differential expression analysis (Hughes et al. 2026). Gene‐level differential expression was analyzed using the dream linear mixed‐model framework in the variancePartition package (Hoffman and Roussos 2020). TMM normalization and voom precision weights were applied before fitting the model: ~ batch + disease + (1 | donor_id). Batch and disease status were modeled as fixed effects, and donor identity was included as a random intercept to account for the two repeated wells from each donor. HLF (healthy) samples were the reference group, so the disease coefficient represented IPF relative to HLF fibroblasts. Empirical Bayes moderation was applied, and *p* values were adjusted using the Benjamini–Hochberg method. As a sensitivity analysis, counts from repeated wells were summed for each donor and analyzed using DESeq2 with a design of ~ disease. Batch was not included in this donor‐aggregated model. Metadata for these analyses can be found in “Table S4” and “Table S5” and DEG lists can be found in “Data S7 and S8.”

For targeted visualization in Figure S3B, antiviral‐response genes were grouped a priori into four functional categories based on published reviews of viral RNA sensing and interferon signaling. Viral sensing/proximal signaling included IFIH1 (MDA5), MAVS, and TBK1, reflecting the RIG‐I‐like receptor–MAVS–TBK1 signaling axis that links cytosolic viral RNA recognition to interferon induction (Ivashkiv and Donlin 2014). Transcriptional regulators included IRF3, IRF7, STAT1, and STAT2; IRF3/IRF7 regulate type I interferon induction downstream of pattern‐recognition receptors, whereas STAT1 and STAT2 form part of the interferon‐stimulated gene factor 3 (ISGF3) complex that drives interferon‐stimulated gene transcription (Honda and Taniguchi 2006). Antiviral/IFN‐response effectors included IFIT1–3, MX1, OAS1–3, OASL, ISG15, RSAD2, BST2, and CXCL10, representing downstream interferon‐responsive genes with antiviral restriction or immune‐effector functions (Schneider et al. 2014). Negative regulators included USP18, SOCS1, SOCS3, and PIAS1–4, which negatively regulate interferon/JAK–STAT signaling through complementary feedback mechanisms (Shuai and Liu 2005). Functional grouping was used only to organize the heatmap and did not influence differential‐expression testing.

### 4.12 Transcription Factor Network Analysis

Transcription factor activity was inferred using the univariate linear model implemented in decoupleR (Müller‐Dott et al. 2023). For consistency with the original analysis, CollecTRI interactions annotated by TRRUST as repressive were assigned a mode of regulation of −1, whereas interactions without an explicitly recognized repressive annotation, including interactions with missing or ambiguous directional information, were assigned +1. ULM was applied to shrunken DESeq2 log2 fold changes for the displayed transcription factor activity and target‐gene plots; analyses using DESeq2 Wald statistics were retained as secondary outputs. Because this preprocessing produces a predominantly positively signed regulatory network and ULM infers activity indirectly from coordinated expression of curated target genes, the resulting scores should be interpreted as exploratory predictions that require functional validation.

### 4.13 Gene Set Enrichment Analysis

Preranked gene set enrichment analysis was performed separately for each DESeq2 contrast using the fgsea package (Subramanian et al. 2005). Genes were ranked by the corresponding DESeq2 Wald statistic, with positive and negative values indicating enrichment toward the positive and negative ends of the contrast‐specific expression ranking, respectively. Ensembl identifiers were mapped to gene symbols, and when multiple Ensembl identifiers mapped to the same symbol, the entry with the largest absolute Wald statistic was retained. All eligible gene sets from the human MSigDB Hallmark, Gene Ontology Biological Process (GO:BP), and Reactome collections were tested using gene‐set size limits of 15–500 genes (Liberzon et al. 2015; Aleksander et al. 2023; Milacic et al. 2023). Gene sets were obtained from MSigDB version 2026.1.Hs using msigdbr, and enrichment was calculated using the multilevel implementation of fgsea with scoreType = “std” and eps = 0 (Korotkevich et al. 2021).

Normalized enrichment scores (NES), nominal *p* values, Benjamini–Hochberg false‐discovery‐rate‐adjusted *p* values, and leading‐edge genes were retained for each pathway. Multiple‐testing correction was performed across all eligible pathways within each collection and contrast, and pathways with an adjusted *p* value below 0.05 were considered significantly enriched. The complete Hallmark, GO:BP, and Reactome collections were analyzed before any pathway‐focused visualization to avoid restricting the initial analysis to a predetermined biological response. Antiviral and innate‐immune pathways and metabolic, mitochondrial, and cellular‐stress pathways were subsequently selected from the complete enrichment results using prespecified biological themes for focused visualization and interpretation. These focused pathway panels were used only for presentation; all tested pathways and collection‐wide adjusted *p* values were retained in the accompanying results tables. GSEA results were interpreted as coordinated transcript‐level changes in pathway‐associated genes and not as direct measurements of pathway activity, metabolic flux, or protein function.

### 4.14 Weighted Gene Correlation Network Analysis (WGCNA)

WGCNA was run using pyWGCNA (Rezaie et al. 2023). The topological overlap matrix similarity dendrogram was made using the R WGCNA package and the gene matrices were made using the BioMart R package (Langfelder and Horvath 2008). The gene expression matrix used for pyWGCNA was batch‐corrected and run through a variance stabilizing transformation which was then filtered for the top 75% most varying genes. Resulting gene modules were tested for gene set enrichment analysis (GSEA) against Gene Ontology (GO) Terms, as previously described, and cross‐referenced with DEGs (Ashburner et al. 2000). Our total sample number did not reach the commonly recommended sample number for sufficient power, which is 15 total samples, but meaningful results can still be discovered with less than 15 samples and emphasizes the importance of validating WGCNA module genes regardless of sample size (Langfelder and Horvath 2008; de Silva et al. 2022). WGCNA module gene and DEG overlap lists provided in “Data S2, S3, S5, S6” tables.

### 4.15 Western Blot

For protein extraction, cells were lysed with RIPA buffer supplemented with Halt Protease and Phosphatase Inhibitor (Thermo Fisher Scientific), followed by protein quantification via Micro BCA Protein Assay Kit (Thermo Fisher Scientific). Protein separation was conducted on a 12% SDS‐polyacrylamide gel at 30 mA for 60 min, followed by a transfer to polyvinylidene fluoride membranes at 150 mA for 2 h. Membranes were blocked for 1 h at RT with 5% milk‐Tris‐buffered saline with Tween‐20 (TBST) and then incubated overnight at 4°C with primary antibodies (IRF3 (D83B9) and phospho‐IRF3 (Ser396) (4D4G) rabbit monoclonal antibodies, as well as STAT1 and phospho‐STAT1 (Tyr701) (58D6) antibodies; all used at 1:1000 dilution, Cell Signaling Technology, Danvers, MA, USA).

Secondary HRP‐conjugated antibodies were diluted 1:1000 in 5% milk‐TBST and incubated for 1 h at room temperature. β‐Actin and HRP‐Vinculin were utilized as housekeeping proteins. Membrane images were captured using the iBright CL750 Imaging System (Thermo Fisher Scientific) and quantified with ImageJ. Protein concentrations were normalized to the housekeeping protein and expressed relative to the respective NT control.

### 4.16 Statistical Analysis and Illustrations

Statistical analyses were conducted using GraphPad Prism 10. The specific statistical tests employed are detailed within the corresponding figure legends. Illustrations were created with the aid of Biorender.com.

## Author Contributions

Conceptualization: J.‐W.B.H., P.‐Y.D., S.D.‐E.; methodology: F.H., Y.R., J.‐W.B.H; Cell provision: J.‐W.B.H., A.L.C., T.‐C.H., C.J.L.S.; bioinformatics: J.‐W.B.H., T.A.U.H., F.S., K.S.; writing – original draft preparation: J.‐W.B.H., P.‐Y.D., S.D.‐E., Y.R.; writing – review and editing, all authors; visualization: J.‐W.B.H., P.‐Y.D., S.D.‐E., Y.R.; resources and supervision: S.D.‐E., P.‐Y.D., D.F., C.M.H., C.J.L.S.; funding acquisition, S.D.‐E., P.‐Y.D. All authors have read and agreed to the published version of the manuscript.

## Funding

This work was supported in part by the following grants: NIA P01AG066591, Training Grant—NIA T32AG052374, and BMFTR (Bundesministerium für Forschung, Technologie und Raumfahrt)—13N15745.

## Conflicts of Interest

The authors declare no conflicts of interest.

## Supporting information


**Data S1:** acel70763‐sup‐0001‐Supinfo1.csv.

**Data S2:** acel70763‐sup‐0002‐Supinfo2.csv.

**Data S3:** acel70763‐sup‐0003‐Supinfo3.csv.

**Data S4:** acel70763‐sup‐0004‐Supinfo4.csv.

**Data S5:** acel70763‐sup‐0005‐Supinfo5.csv.

**Data S6:** acel70763‐sup‐0006‐Supinfo6.csv.

**Data S7:** acel70763‐sup‐0007‐Supinfo7.csv.

**Data S8:** acel70763‐sup‐0008‐Supinfo8.csv.

**Figure S1:** (A) Gene set enrichment analysis (GSEA) wheel plot for quiescent (QUI) healthy (HLF) and IPF lung fibroblasts without infection (mock; left). Gene set enrichment analysis (GSEA) wheel plots for quiescent (QUI) healthy (HLF) and IPF lung fibroblasts without mock infection using dream (middle) or DESeq2 analysis (right). Middle and right wheel plots data gathered from GEO Super Series GSE328437 RNA‐seq data GSE328392. Wheel plots are a predefined selection of viral‐associated pathways after running GSEA against the complete MSigDB Hallmark, Gene Ontology Biological Process, and Reactome collections. (B) Top activated and repressed transcription factors (TF) inferred by decoupleR (ULM) using CollecTRI and DESeq2 log2FC Wald statistics for QUI HLF and IPF lung fibroblasts without infection (mock). Bars indicate TF activity scores (red, activated; blue, repressed).
**Figure S2:** (A) Viral titers in the supernatant of human lung fibroblasts (IMR‐90 cells) infected with influenza A virus (IAV) for 8 and 24 h were determined via plaque assay (*n* = 5). Viral load was quantified as plaque‐forming units (PFU) per ml and data is displayed on a logarithmic axis. (B) Log2 fold change of several interferon‐stimulated genes (ISGs) for IAV infected vs. mock IMR‐90 cells at 8 and 24 h post infection (hpi), determined by bulk RNA sequencing of three biological replicates per group. Displayed genes were significantly differentially expressed based on *p*‐value. Statistical significance was calculated using Mann–Whitney U test (A). **p < 0.01.
**Figure S3:** (A) Gene set enrichment analysis (GSEA) wheel plot for senescent (SEN) healthy (HLF; left) and IPF (right) lung fibroblasts after infection (IAV). Wheel plots are a predefined selection of viral‐associated pathways after running GSEA against the complete MSigDB Hallmark, Gene Ontology Biological Process, and Reactome collections. (B) Heatmap of unique genes between quiescent (QUI) HLF and IPF lung fibroblasts without infection and SEN HLF and IPF lung fibroblasts after infection (IAV). Genes are grouped into an a priori selected antiviral‐response gene panel grouped into four functional categories.
**Figure S4:** (A, B) Gene set enrichment analysis (GSEA) wheel plot for senescent (SEN) healthy (HLF) and IPF (right) lung fibroblasts after infection (IAV). Wheel plots are a predefined selection of publicly available senescence‐associated pathway gene lists. Wheel plots are split into enrichment in upregulated (left) versus downregulated (right) genes. (C) Gene set enrichment analysis (GSEA) wheel plot for mock‐infected, quiescent (QUI) HLF and IPF lung fibroblasts (left) and SEN HLF (middle) and IPF (right) lung fibroblasts after infection (IAV). Wheel plots are a predefined selection of metabolism‐associated pathways after running GSEA against the complete MSigDB Hallmark, Gene Ontology Biological Process, and Reactome collections.
**Figure S5:** Intracellular viral RNA levels, expressed as copies/μl, were quantified by qRT‐PCR in quiescent (QUI) and senescent (SEN) healthy (HLF) and idiopathic pulmonary fibrosis (IPF) lung fibroblasts (*n* = 3–4). Data is displayed on a logarithmic axis. Statistical significance was determined using the Mann–Whitney U test. * *p* < 0.05.
**Figure S6:** (A, B) Relative quantification of IRF3 and p‐IRF3 protein levels (A) as well as STAT1 and p‐STAT1 protein levels (B) in senescent (SEN) healthy lung fibroblasts (HLF) via Western blot (*n* = 2). Protein levels were quantified using Image J and are normalized to the respective housekeeping protein (Vinculin for IRF3/p‐IRF3 and β‐Actin for STAT1/p‐STAT1) and are expressed relative to the respective NT control siRNA condition. (C) The relative expression of senescence markers CDKN1A (p21), CDKN2A (p16), and LMNB1 (Lamin B1) was quantified by qRT‐PCR in both infected quiescent (QUI) and SEN HLF and idiopathic pulmonary fibrosis (IPF) fibroblasts transfected with NT or IRF3 siRNA (*n* = 4). Gene expression levels were normalized to β‐Actin and reported relative to the respective QUI control. Statistical significance was determined using the Mann–Whitney U test (C). * *p* < 0.05.
**Figure S7:** (A–D) Relative expression of the interferon‐stimulated genes (ISGs) MX1 (A), ISG15 (B), IFIT1 (C), and OAS1 (D) was quantified by qRT‐PCR in infected quiescent (QUI) and senescent (SEN) fibroblasts. Healthy lung fibroblasts (HLF) and idiopathic pulmonary fibrosis (IPF) fibroblasts were transfected with nontargeting (NT), IRF3, or STAT1 siRNA prior to infection (*n* = 4). Gene expression levels were normalized to β‐Actin and reported relative to the respective mock control. Statistical significance was determined using the Mann–Whitney U test (A‐D). * *p* < 0.05.
**Figure S8:** (A, B) Principal component analysis (PCA) of non‐batch corrected (left) and batch corrected (right) quiescent (QUI) and senescent (SEN) HLF and IPF lung fibroblasts with mock or IAV treatment. (C, D) DESeq2 results from models without batch adjustment (left) versus models including batch (right) as a covariate from HLF SEN IAV lung fibroblasts. DEG cutoffs were defined by log2FC < −0.5 or > 0.5 and p‐adjusted value < 0.05. Top 20 DEGs labeled and the volcano plot was capped at −5 to 5 log2FC.
**Table S1:** acel70763‐sup‐0009‐Supinfo9.zip.
**Table S2:** Forward and reverse primer sequences used in qRT‐PCR. All primers are human.
**Table S3:** acel70763‐sup‐0009‐Supinfo9.zip.
**Table S4:** acel70763‐sup‐0009‐Supinfo9.zip.
**Table S5:** acel70763‐sup‐0009‐Supinfo9.zip.

## Data Availability

Raw sequencing data (FASTQ files) and processed bulk RNA‐seq have been deposited in the Gene Expression Omnibus (GEO) under Series record accession GSE334185. Metadata can be found in “Table S3.” Additional data that support the findings of this study are available from the corresponding author upon reasonable request. All analysis code, figure‐generation notebooks and workflow utilities used in this study are available in the GitHub repository: https://github.com/jwbrendan78/ipf‐senescence‐antiviral‐rnaseq.
