# Supplementary material for: Cellular Senescence Dysregulates Antiviral Interferon Responses in Idiopathic Pulmonary Fibrosis
Source: Aging Cell. 2026 Oct 20;25(11):e70763. doi: 10.1111/acel.70763 (PMC13684319; doi:10.1111/acel.70763)
Supplement: Supplementary file 1 [file ACEL-25-e70763-s004.zip › 20260902_Buck Jena paper_supplemental.docx]

# **Supplementary Figure 1**

(A) Gene set enrichment analysis (GSEA) wheel plot for quiescent (QUI) healthy (HLF) and IPF lung fibroblasts without infection (mock; left). Gene set enrichment analysis (GSEA) wheel plots for quiescent (QUI) healthy (HLF) and IPF lung fibroblasts without mock infection using dream (middle) or DESeq2 analysis (right). Middle and right wheel plots data gathered from GEO Super Series GSE328437 RNA-seq data GSE328392. Wheel plots are a predefined selection of viral-associated pathways after running GSEA against the complete MSigDB Hallmark, Gene Ontology Biological Process, and Reactome collections. (B) Top activated and repressed transcription factors (TF) inferred by decoupleR (ULM) using CollecTRI and DESeq2 log2FC Wald statistics for QUI HLF and IPF lung fibroblasts without infection (mock). Bars indicate TF activity scores (red, activated; blue, repressed).

# **Supplementary Figure 2**

(A) Viral titers in the supernatant of human lung fibroblasts (IMR-90 cells) infected with influenza A virus (IAV) for 8 and 24 hours were determined via plaque assay (n = 5). Viral load was quantified as plaque-forming units (PFU) per ml and data is displayed on a logarithmic axis. (B) Log_2_ fold change of several interferon-stimulated genes (ISGs) for IAV infected vs. mock IMR-90 cells at 8 and 24 hours post infection (hpi), determined by bulk RNA sequencing of three biological replicates per group. Displayed genes were significantly differentially expressed based on p-value. Statistical significance was calculated using Mann-Whitney U test (A). **p < 0.01.

# **Supplementary Figure 3**

(A) Gene set enrichment analysis (GSEA) wheel plot for senescent (SEN) healthy (HLF; left) and IPF (right) lung fibroblasts after infection (IAV). Wheel plots are a predefined selection of viral-associated pathways after running GSEA against the complete MSigDB Hallmark, Gene Ontology Biological Process, and Reactome collections. (B) Heatmap of unique genes between quiescent (QUI) HLF and IPF lung fibroblasts without infection and SEN HLF and IPF lung fibroblasts after infection (IAV). Genes are grouped into an a priori selected antiviral-response gene panel grouped into four functional categories.

# **Supplementary Figure 4**

(A, B) Gene set enrichment analysis (GSEA) wheel plot for senescent (SEN) healthy (HLF) and IPF (right) lung fibroblasts after infection (IAV). Wheel plots are a predefined selection of publicly available senescence-associated pathway gene lists. Wheel plots are split into enrichment in upregulated (left) versus downregulated (right) genes. (C) Gene set enrichment analysis (GSEA) wheel plot for mock-infected, quiescent (QUI) HLF and IPF lung fibroblasts (left) and SEN HLF (middle) and IPF (right) lung fibroblasts after infection (IAV). Wheel plots are a predefined selection of metabolism-associated pathways after running GSEA against the complete MSigDB Hallmark, Gene Ontology Biological Process, and Reactome collections.

# **Supplementary Figure 5**

Intracellular viral RNA levels, expressed as copies/µl, were quantified by qRT-PCR in quiescent (QUI) and senescent (SEN) healthy (HLF) and idiopathic pulmonary fibrosis (IPF) lung fibroblasts (n = 3–4). Data is displayed on a logarithmic axis. Statistical significance was determined using the Mann-Whitney U test. * p < 0.05.

# **Supplementary Figure 6**

(A, B) Relative quantification of IRF3 and p-IRF3 protein levels (A) as well as STAT1 and p-STAT1 protein levels (B) in senescent (SEN) healthy lung fibroblasts (HLF) via Western blot (n = 2). Protein levels were quantified using Image J and are normalized to the respective housekeeping protein (Vinculin for IRF3/ p-IRF3 and β-Actin for STAT1/ p-STAT1) and are expressed relative to the respective NT control siRNA condition. (C) The relative expression of senescence markers *CDKN1A* (p21), *CDKN2A* (p16), and *LMNB1* (Lamin B1) was quantified by qRT-PCR in both infected quiescent (QUI) and SEN HLF and idiopathic pulmonary fibrosis (IPF) fibroblasts transfected with NT or *IRF3* siRNA (n = 4). Gene expression levels were normalized to β-Actin and reported relative to the respective QUI control. Statistical significance was determined using the Mann-Whitney U test (C). * p < 0.05.

# **Supplementary Figure 7**

(A-D) Relative expression of the interferon-stimulated genes (ISGs) *MX1* (A), *ISG15* (B), *IFIT1* (C), and *OAS1* (D) was quantified by qRT-PCR in infected quiescent (QUI) and senescent (SEN) fibroblasts. Healthy lung fibroblasts (HLF) and idiopathic pulmonary fibrosis (IPF) fibroblasts were transfected with non-targeting (NT), *IRF3*, or *STAT1* siRNA prior to infection (n = 4). Gene expression levels were normalized to β-Actin and reported relative to the respective mock control. Statistical significance was determined using the Mann-Whitney U test (A-D). * p < 0.05.

# **Supplementary Figure 8**

(A, B) Principal component analysis (PCA) of non-batch corrected (left) and batch corrected (right) quiescent (QUI) and senescent (SEN) HLF and IPF lung fibroblasts with mock or IAV treatment. (C, D) DESeq2 results from models without batch adjustment (left) versus models including batch (right) as a covariate from HLF SEN IAV lung fibroblasts. DEG cutoffs were defined by log2FC < -0.5 or > 0.5 and p-adjusted value < 0.05. Top 20 DEGs labeled and the volcano plot was capped at -5 to 5 log2FC.

# **Supplementary Table 2**

| **Gene name** | **Forward sequence** | **Reverse sequence** |
| --- | --- | --- |
| *IRF3* | TCTGCCCTCAACCGCAAAGAAG | TACTGCCTCCACCATTGGTGTC |
| *STAT1* | ATGGCAGTCTGGCGGCTGAATT | CCAAACCAGGCTGGCACAATTG |
| *ACTB* | CATGTACGTTGCTATCCAGGC | CTCCTTAATGTCACGCACGAT |
| *LMNB1* | TTGGATGCTCTTGGGGTTC | AAGCAGCTGGAGTGGTTGTT |
| *CDKN1A* (p21) | TCACTGTCTTGTACCCTTGTGC | GGCGTTTGGAGTGGTAGAAA |
| *CDKN2A* (p16) | CTCGTGCTGATGCTACTGAGGA | GGTCGGCGCAGTTGGGCTCC |
| *MX1* | GGTGGTCCCCAGTAATGTGG | CGTCAAGATTCCGATGGTCCT |
| *ISG15* | GTGGACAAATGCGACGAACC | TCGAAGGTCAGCCAGAACAG |
| *IFIT1* | TGACTCTTTTGCCTCTTTCTTCTAA | TTCTTGGGGTGCTCTGTGG |
| *OAS1* | AGTTGACTGGCGGCTATAAAC | GTGCTTGACTAGGCGGATGAG |

Forward and reverse primer sequences used in qRT-PCR. All primers are human.
